# Longitudinal changes in quality of life among patients receiving home nutrition support: a systematic review and meta-analysis

**DOI:** 10.3389/fnut.2026.1940328

**Published:** 2026-10-19

**Authors:** Wenjing Guo, Chulin Chen, Yang Yang, Juan Ni, Xinying Wang

**Affiliations:** 1 School of Nursing, Nanjing University of Chinese Medicine, Nanjing, Jiangsu, China; 2 Research Institute of General Surgery, Jinling Hospital, Affiliated Hospital of Medical School, Nanjing University, Nanjing, China; 3 Jinling Clinical Medical College, Nanjing University of Chinese Medicine, Nanjing, China

**Keywords:** home enteral nutrition, home nutrition support, home parenteral nutrition, meta-analysis, quality of life

## Abstract

**Background:**

Home nutrition support (HNS), including home enteral nutrition (HEN) and home parenteral nutrition (HPN), is widely used in patients who cannot meet their nutritional requirements through oral intake alone. However, longitudinal changes in quality of life (QoL) following HNS have not been systematically synthesized.

**Methods:**

PubMed, Embase, Web of Science, CNKI, Wang fang, VIP, SinoMed, and the Cochrane Library were searched from inception to June 2026 to identify longitudinal studies evaluating QoL among adults receiving HNS. The methodological quality of included studies was assessed. QoL changes at 1, 2, 3, 4, and 6 months after HNS initiation were compared with baseline and synthesized using meta-analysis.

**Results:**

Eighteen studies were included. The pooled SMDs at 1, 2, 3, 4, and 6 months were 0.29, 0.54, 0.64, 0.61, and 0.09, respectively. The 1 to 4 month estimates were statistically significant, while the 6 month estimate was not (95% CI: −0.20 to 0.38). Descriptive evidence from three non-pooled studies suggested possible improvements in the physical and psychosocial domains of QoL.

**Conclusions:**

Patients receiving HNS experienced statistically significant improvements from baseline in QoL during the first 1–4 months, with the largest pooled estimates observed between 2 and 4 months. The improvement was smaller at 6 months and did not reach statistical significance. The pattern suggests a descriptive temporal trend, though the studies contributing to each time point differed. Further longitudinal studies with longer follow-up are needed to clarify the long-term changes of QoL among patients receiving HNS.

**Systematic review registration:** https://www.crd.york.ac.uk/prospero/display_record.php?ID=CRD420261414696, identifier: CRD420261414696.

## 1 Introduction

Home nutrition support (HNS)-comprising home enteral nutrition (HEN) and home parenteral nutrition (HPN)-has become essential for patients unable to meet nutritional needs orally. HEN delivers enteral nutrition at home via oral supplements or tube feeding (e.g., nasogastric, gastrostomy, or jejunostomy tubes), typically initiated in hospital and continued long-term (1). In contrast, HPN provides parenteral nutrition through intravenous access, serving as a life-sustaining alternative for those who cannot tolerate oral or enteral intake, particularly patients with chronic intestinal failure (2). Driven by the popularization of early discharge and home-based care, the application of HNS has grown rapidly across the globe. Current research pays more attention to patients' long-term physical and mental health rather than merely short-term survival. Quality of life (QoL) has therefore become a vital indicator for comprehensively evaluating the outcomes of nutritional support (2, 3).

QoL is a multidimensional concept encompassing physical functioning, psychological wellbeing, social participation, symptom burden, and overall satisfaction with life (4). This outcome is particularly relevant for patients receiving HNS, as nutritional support often extends over prolonged periods and requires adaptation to feeding devices, self-management responsibilities, and lifestyle changes. Consistent evidence from previous studies indicates that HNS can significantly improve patients' physical energy, physical function, daily independence, and overall QoL by optimizing nutritional status, alleviating symptoms, and promoting functional recovery (5–7).

However, the relationship between HNS and QoL remains complex. While nutritional support may alleviate symptoms and improve physical health, patients frequently encounter challenges associated with long-term treatment. Qualitative studies have shown that individuals receiving HNS often experience sleep disturbances, restrictions in social participation, altered family relationships, body image concerns, and substantial treatment-related burdens (8, 9). In addition, caregivers commonly report emotional stress, increased caregiving responsibilities, and reduced QoL associated with home nutritional care (10). Consequently, HNS may generate both benefits and burdens, resulting in heterogeneous QoL outcomes across different patient populations and stages of treatment. Consistent with this complexity, previous studies have reported conflicting findings, with some demonstrating significant improvements in overall QoL, whereas others have observed limited benefits or deterioration in specific aspects: many patients develop anxiety and depressive symptoms, suffer persistent gastrointestinal discomfort, and experience chronic sleep disorders induced by overnight nutrient infusion, which collectively impair their health-related QoL (11, 12). Variations in disease characteristics, nutritional support modalities, and assessment instruments may partially explain these discrepancies (13).

Although systematic reviews have evaluated the clinical outcomes of HNS (14, 15), longitudinal changes in QoL across different follow-up periods have not been comprehensively synthesized. Understanding these changes is important for identifying critical periods of adaptation, optimizing nutritional management, and developing targeted supportive interventions. Therefore, this systematic review and meta-analysis aimed to evaluate longitudinal changes in QoL among patients receiving HNS and to examine whether QoL changes differed according to the type of HNS and QoL assessment instruments.

## 2 Materials and methods

This study was reported under the Preferred Reporting Items for Systematic Review and Meta-analyses (PRISMA) reporting guidelines (16). We have registered this meta-analysis on PROSPERO, and the information is available on http://www.crd.york.ac.uk/PROSPERO/display~record.asp?ID=CRD420261414696.

### 2.1 Search strategy

A systematic literature search was conducted in PubMed, Embase, Web of Science, CNKI, Wang fang, VIP, SinoMed, and the Cochrane Library from database inception to June 2026. The search strategy combined Medical Subject Headings (Mesh) and free-text terms related to home nutrition support and quality of life. The main search terms included “home nutrition support,” “home enteral nutrition,” “home parenteral nutrition,” “quality of life,” “health-related quality of life” (Supplementary Material S1). Reference lists of relevant reviews and included studies were also manually searched to identify additional eligible articles.

### 2.2 Eligibility criteria

Studies were considered eligible if they met the following criteria: (1) participants were adults (≥18 years) receiving HNS, including HEN and/or HPN; (2) QoL was assessed using validated instruments, such as the SF-36, SF-12, EQ-5D, EORTC QLQ-C30, WHOQOL-BREF, or other validated QoL scales (17); (3) studies employed a longitudinal design, including randomized controlled trials, quasi-experimental studies, prospective longitudinal observational studies, or case-series studies with repeated follow-up assessments; (4) QoL outcomes were reported at baseline and at least one follow-up time point after initiation of HNS; (5) sufficient statistical information was available to calculate effect sizes; (6) studies were published in either English or Chinese.

Studies were excluded if they: (1) involved pediatric populations; (2) used a cross-sectional design without repeated measurements; (3) did not report QoL outcomes; (4) were reviews, editorials, conference abstracts, study protocols, case reports; or (5) lacked sufficient data for effect size calculation.

### 2.3 Study selection and data extraction

All retrieved records were imported into EndNote software, and duplicate records were removed. Two reviewers independently screened titles and abstracts for eligibility. Full texts of potentially relevant studies were subsequently assessed according to the predefined inclusion and exclusion criteria. Any disagreements were resolved through discussion or consultation with a third reviewer.

Data extraction was performed independently by two reviewers using a standardized data extraction form. For randomized controlled trials, only data from the intervention group (patients receiving HNS) were extracted, as our analysis focused on the longitudinal changes in QoL within HNS–treated populations rather than between–group comparative effects. The following information was collected: first author, publication year, country, study design, sample size, participant characteristics, underlying disease, type of HNS, QoL assessment instrument, follow-up duration, and outcome data. For each time point, mean scores, standard deviations, and sample sizes were extracted. When multiple follow-up assessments were reported, data were extracted separately for each available time point. The mean and standardized differences were used to calculate the standardized mean difference (SMD) and the standard error (SE).

### 2.4 Study quality assessment

We utilized the Joanna Briggs Institute (JBI) critical appraisal tools for the quality assessment of eligible articles (18). Methodological quality was assessed independently by two reviewers using the Joanna Briggs Institute (JBI) Critical Appraisal Tools according to study design. The JBI Checklist for Case Series was used for longitudinal single-arm studies, the JBI Checklist for Randomized Controlled Trials was applied to randomized studies, and the JBI Checklist for Quasi-Experimental Studies was used for non-randomized intervention studies. Each item was rated as “Yes,” “No,” “Unclear,” or “Not Applicable,” as appropriate. The detailed content of each JBI checklist item is provided in Supplementary Table S1. Disagreements were resolved through discussion and consultation with a third reviewer when necessary. In addition to the JBI scores, we extracted the following information for each single–arm study to better contextualize methodological quality: attrition rates and documentation of reasons for dropout; handling of missing outcome data (e.g., imputation methods or complete–case analysis); and whether any adjustments for confounding or within–subject correlation were applied. These parameters were considered alongside the JBI scores when interpreting the robustness of individual study contributions to the pooled estimates.

### 2.5 Statistical analysis

A generic inverse variance method was used to pool standardized mean differences (SMDs) in quality-of-life (QoL) scores between baseline and follow-up assessments. The SMD was selected as the summary effect measure because different instruments were used to assess QoL across studies (19). SE required for inverse-variance weighting were not consistently reported; therefore, SEs and SMDs were calculated according to the Cochrane Handbook (19) and Borenstein et al. (20), and missing change data were derived from reported *t*-values, *p*-values, or 95% confidence intervals when available. For each study, the within-group change from baseline to each follow-up was calculated as Meanfollow-up—Meanbaseline, and the standard deviation of the change was imputed using the formula $ChangeSD=\sqrt{{SD^{2}}_{baseline}{{+SD}^{2}}_{followup}{{-2r\times SD}^{2}}_{baseline}{\times SD}_{followup}}.$ Because correlations between baseline and follow-up measurements were not reported in most studies, a correlation coefficient of r = 0.50 was assumed for the primary analysis, with *r* = 0.10 and *r* = 0.90 used in sensitivity analyses to assess robustness. To ensure that a positive SMD consistently indicated improvement, we confirmed that all QoL instruments included in the meta–analysis were scored in the same direction (higher scores = better QoL); therefore, no reverse scoring was applied. For longitudinal synthesis, follow–up assessments were assigned to five predefined time windows (1, 2, 3, 4, and 6 months) using a rounding rule: assessments at ≤ 6 weeks were assigned to 1 month; those at >6 to ≤ 10 weeks to 2 months; those at >10 to ≤ 14 weeks to 3 months; those at >14 to ≤ 20 weeks to 4 months; and those at >20 to ≤ 28 weeks to 6 months. When more than one assessment fell within the same interval, the assessment closest to the nominal time point was used. Studies with follow–up outside these windows were not included in the longitudinal synthesis. Formal publication bias analyses were not performed due to the limited number of studies (*n* < 10) in most time-point subgroups, as recommended by the Cochrane Handbook (19). Robustness was instead assessed via sensitivity analyses.

We chose a random-effects model to analyze the data. The *I*^2^ value identified statistical heterogeneity between the studies; *I*^2^ ≥ 50% indicated high statistical heterogeneity. Subgroup analyses were conducted based on nutritional support methods and QoL measurement instruments to explore potential sources of heterogeneity; sensitivity analyses were performed alongside variation of the assumed correlation coefficient. All statistical analyses were performed using R software (version 4.5.2 Foundation for Statistical Computing, Vienna, Austria), with a two-sided *P* value < 0.05 considered statistically significant.

## 3 Results

### 3.1 Study selection

A total of 3,525 records were identified through database searching. After removal of duplicates, 2,370 records underwent title and abstract screening, of which 122 articles were retrieved for full-text review. Following full-text assessment, 104 studies were excluded because they were cross-sectional studies or randomized controlled trials with only a single assessment point (*n* = 33), provided insufficient data for effect size calculation (*n* = 44), involved ineligible populations (*n* = 14), were conference abstracts (*n* = 6), were published in languages other than English or Chinese (*n* = 4), or had unavailable full texts (*n* = 3). A detailed list of the 44 excluded studies with specific reasons is provided in Supplementary Table S2. The missing data primarily included absence of baseline QoL scores, data reported as medians (IQR) rather than mean ± SD, only subscale scores without a global/total QoL score, and use of performance status instruments (e.g., KPS) rather than standardized patient-reported QoL measures. Three additional studies met the inclusion criteria for the systematic review but could not be pooled quantitatively because they did not report a compatible overall/global QoL score. Zeng et al. and Huang et al. reported only dimension-specific QoL scores without a global/total score, whereas Pinto-Sánchez et al. (33) reported only domain-specific change-from-baseline values for the SF-36, without a compatible overall/global QoL score or endpoint scores. Therefore, consistent effect-size calculation across studies was not possible. Ultimately, 18 studies were included in the systematic review, and 15 of these were eligible for the final meta-analysis (21–38). The study selection process is shown in Figure 1.

**Figure 1:**
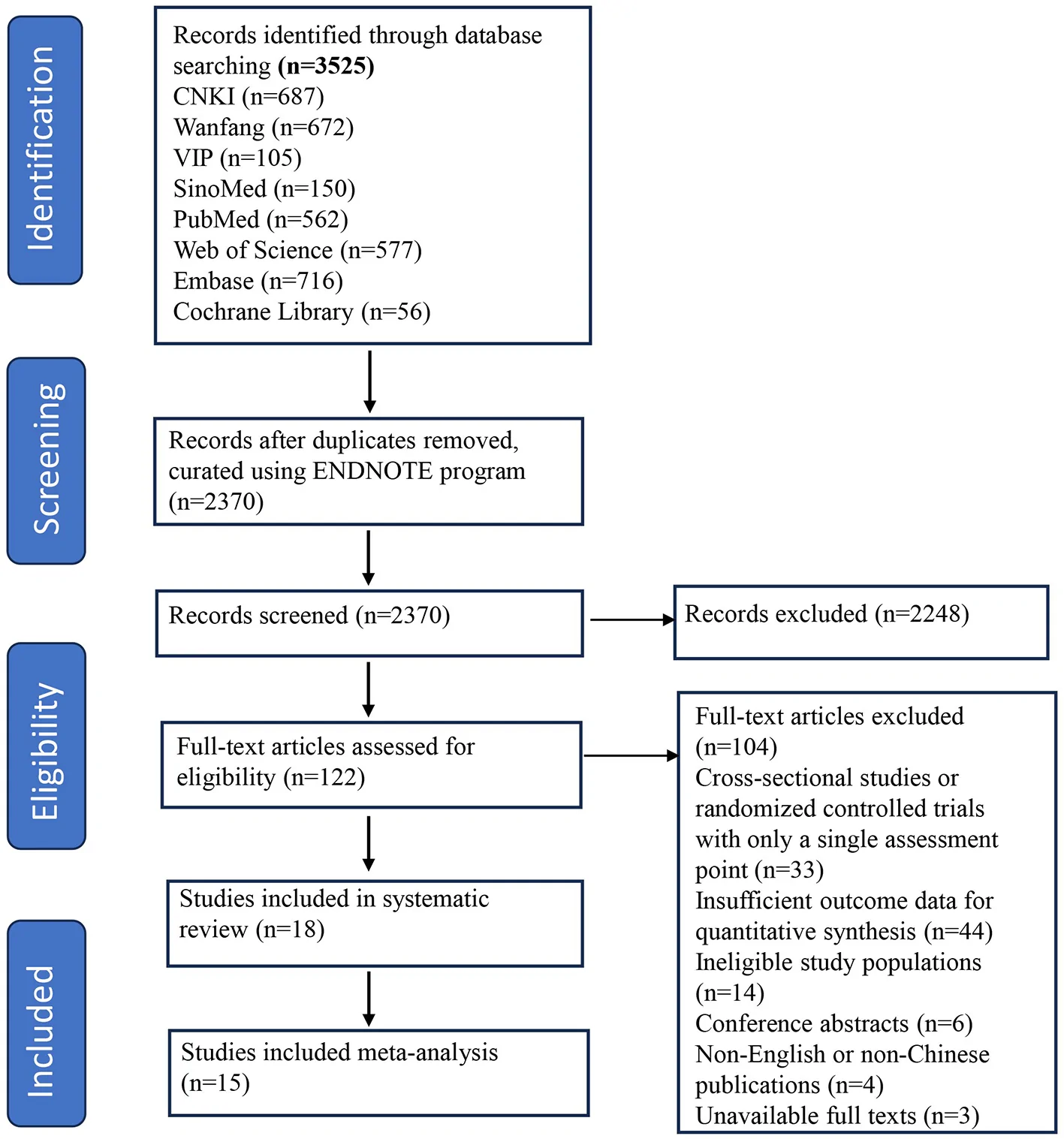
PRISMA flow diagram.

### 3.2 Characteristics of included studies

The 18 included studies, published between 2000 and 2025, were conducted across Asia, Europe, and North America, with 7 studies from China, 3 from France, 2 from Spain, and 1 each from the United Kingdom, Italy, Germany, Denmark, Canada and the United States. Among the included studies, 11 investigated HEN and 7 investigated HPN. Various validated QoL instruments were used across the included studies, with EORTC QLQ-C30 being the most frequently applied measure. Most studies conducted repeated follow-up assessments within 6 months after initiation of HNS, with 1, 3, and 6-month follow-up time points reported most commonly. The route and mode of nutrition support varied across studies. In HEN studies, the nasogastric tube was the most common route, followed by gastrostomy, jejunostomy, and oral nutritional supplements, with some studies including mixed routes. For HPN, all patients received nutrition via central venous catheters, although some studies did not report these details. Detailed study characteristics are presented in Table 1.

**Table 1:** The characteristics of the included studies.

References	Country	Sample size (I/F)	Study design	Disease type	Intervention type/Route	Mode of administration	Mean age	Follow-up time points	Quality of life assessment tool (s)
Cao (21)	China	93/93	Prospective longitudinal study	Crohn's disease patients	HEN/NG tube	Pump-assisted infusion	36.37 ± 11.38	Baseline, 1 month, 3 months	IBDQ
Cui (22)	China	13/13	RCT	Postoperative gastric cancer patients	HEN/ONS	Oral self-administration, 3 times daily between meals	58.2 ± 17.5	Baseline, 1 month, 2 months, 3 months	EQ-5D
Huang (23)	China	156/156	Prospective longitudinal study	Crohn's disease patients	HEN/Mixed (NG tube, NJ tube, PEG/J tube, gastrostomy, jejunostomy, and ONS)	Gravity continuous drip (for tube feeding); oral self-administration (for ONS)	Unclear	Baseline, 1 month, 2 months, 3 months	SF-36
Yang (24)	China	43/43	RCT	Postoperative adjuvant chemotherapy patients with colorectal cancer	HEN/ONS	Oral self-administration	58.0 ± 14.5	Baseline, 1 month, 2 months, 3 months	EQ-5D
Chen (25)	China	288/288	Prospective longitudinal study	Mixed (intestinal motility disorders, IBD, SBS, digestive system malignancy, and others)	HEN/Mixed (oral, NG/NI tube, PEG-J/PEG)	Unclear	45.44 ± 16.27	Baseline, 3 months, 6 months	SF-36
Bowery (26)	UK	54/36	RCT	Postoperative esophageal/gastric cancer	HEN/Jejunostomy tube	Overnight tube feeding via electronic pump	64.6 ± 8.0	Baseline, 6 weeks, 3 months, 6 months	EORTC QLQ-C30/EORTC QLQ-OG25
Cotogni (27)	Italy	97/49	Prospective longitudinal study	Advanced cancer patients	HPN/Central venous catheter	Night-time infusion via electronic pump (10–14 h/day)	Unclear	Baseline, 1 month, 2 months, 3 months, 4 months	EORTC QLQ-C30
Culine (28)	France	437/437	Prospective longitudinal study	Cancer patients	HPN/Central venous catheter	Overnight infusion (84.7% of patients)	63 ± 11.4	Baseline, 28 days	FACT-G
Rocamora (29)	Spain	57/36	Prospective longitudinal study	Patients requiring HEN	HEN/Mixed (oral, NG tube, NJ tube, gastrostomy, jejunostomy)	Unclear	53.8 ± 17.2	Baseline, 3 months, 6 months	NutriQoL
Loeser (30)	Germany	56/56	Prospective longitudinal study	Patients requiring HETF (neurological and oncological diseases)	HEN/PEG tube	Unclear	Unclear	Baseline, 2 months, 4 months	EORTC QLQ-C30
Ma (31)	Taiwan, China	25/12	Prospective longitudinal study	Unresectable/metastatic gastric cancer receiving palliative hemotherapy	HPN/CVC	Daily infusion (18–24 h/day)	68 (35–88)	Baseline, 0.5 month, 1 month, 2 months, 3 months	EORTC QLQ-C30
Obling (32)	Denmark	22/8	RCT	Incurable gastrointestinal cancer	HPN/CVC	Intermittent infusion, 2–4 days/week	66.9 (41.5–88.2)	Baseline, 6 weeks, 12 weeks, 18 weeks, 24 weeks	EORTC QLQ-C15-PAL
Pinto-Sanchez (33)	Canada	21/18	Prospective longitudinal study	Mixed (malignancy 52%, IBD 28%, dysmotility 10%, SBS 7%)	HPN/CVC	Overnight infusion	47.5 (19–70)	Baseline, 1 month, 3 months	SF-36
Senesse (34)	France	205/205	Prospective longitudinal study	Gastrointestinal cancer	HPN/CVC	Overnight infusion	64.5 ± 11.0	Baseline, 28 days	FACT-G
Vashi (35)	US	52/39	Prospective longitudinal study	Advanced cancer patients	HPN/CVC	Daily cyclic infusion	53.2 ± 9.4	Baseline, 1 month, 2 months, 3 months	EORTC QLQ-C30
Zamanillo (36)	Spain	78/64	Prospective longitudinal study	Cancer (89.7%), others (neurological, digestive, etc., 10.3%)	HEN/Mixed (oral 57.7%, tube 42.3%: NG, gastrostomy, jejunostomy)	Oral: self-administration; Tube: syringe, gravity drip, or pump infusion	64.2 ± 10.5	Baseline, 2 months	NutriQoL, F-12
Roberge (37)	France	39/30	Prospective longitudinal study	Head and neck cancer esophageal cancer	HEN/Mixed (NG tube 80%, gastrostomy tube 20%)	Intermittent feeding	58 (38–74)	Baseline, day 28	EORTC QLQ-C30
Zeng (38)	China	30/30	quasi-experimental	Postoperative esophageal cancer	HEN/Jejunostomy tube	Unclear	61.7 ± 8.4	1 week, 4 weeks, 12 weeks, 24 weeks	EORTC QLQ-C30

I/F, initial/final sample size; RCT, randomized controlled trial; HEN, home enteral nutrition; HPN, home parenteral nutrition; ONS, oral nutritional supplements; NG, nasogastric; NJ, nasojejunal; NI, nasointestinal; PEG, percutaneous endoscopic gastrostomy; PEG/J (PEG-J), percutaneous endoscopic gastrostomy/jejunostomy; CVC, central venous catheter; HETF, home enteral tube feeding; IBD, inflammatory bowel disease; SBS, short bowel syndrome; QoL, quality of life; IBDQ, Inflammatory Bowel Disease Questionnaire; EQ-5D, EuroQol 5-Dimension; SF-36, 36-Item Short Form Health Survey; SF-12, 12-Item Short Form Health Survey; EORTC QLQ-C30, European Organization for Research and Treatment of Cancer Quality of Life Questionnaire-Core 30; EORTC QLQ-OG25, EORTC Quality of Life Questionnaire-Oesophagogastric 25; EORTC QLQ-C15-PAL, EORTC Quality of Life Questionnaire-Core 15 Palliative; FACT-G, Functional Assessment of Cancer Therapy-General; NutriQoL, Nutrition Quality of Life questionnaire. Age is presented as mean ± SD or median (range). Unclear indicates not reported or not extractable from the original article. Mixed indicates multiple routes/modes of nutrition intervention; see the original article for details. In the Intervention type/Route column, the value before the slash indicates the nutrition support type, and the value after the slash indicates the specific feeding route or supplementation mode.

### 3.3 Quality assessment

Overall, the methodological quality of the included studies was acceptable. Among the 12 longitudinal observational studies, most clearly defined inclusion criteria, reported participant characteristics, used valid outcome measures, and provided appropriate statistical analyses. However, several studies provided limited information regarding consecutive recruitment and complete inclusion of eligible participants. The 4 randomized controlled trials generally demonstrated adequate randomization procedures and baseline comparability between groups, although blinding of participants and personnel was rarely feasible. The 2 quasi-experimental studies fulfilled most methodological criteria but provided limited information regarding comparison groups and potential sources of bias. Detailed quality assessment results are presented in Tables 2–4. We further extracted three additional parameters—attrition rates, handling of missing data, and confounding control—specifically for the 12 single–arm studies assessed using the JBI Case Series Checklist. Attrition rates varied considerably across studies, ranging from 0% to 71%; most studies did not report methods for handling missing data, and only four studies (25, 27, 35, 36) employed statistical methods to adjust for confounding. A detailed summary is provided in Supplementary Table S3.

**Table 2:** Quality appraisal of included literatures using JBI checklist for case series (10 items, 12 Literatures).

References	Q1	Q2	Q3	Q4	Q5	Q6	Q7	Q8	Q9	Q10
Cao (21)	Y	Y	Y	Y	N	N	Y	Y	U	Y
Huang (23)	N	Y	U	Y	N	N	Y	Y	N	Y
Chen (25)	Y	Y	Y	Y	Y	Y	Y	Y	N	Y
Cotogni (27)	Y	Y	Y	Y	Y	Y	Y	Y	Y	Y
Culine (28)	Y	Y	Y	Y	N	N	Y	Y	U	Y
Irles-Rocamora et al. (29)	Y	Y	N	Y	N	N	Y	Y	U	Y
Loeser (30)	Y	Y	Y	Y	Y	Y	Y	Y	Y	Y
Pinto-Sanchez (33)	Y	Y	Y	Y	N	N	Y	Y	Y	Y
Roberge (37)	Y	Y	Y	Y	N	N	Y	Y	N	Y
Senesse (34)	Y	Y	Y	Y	N	N	Y	Y	N	Y
Vashi (35)	Y	Y	Y	Y	U	Y	Y	Y	Y	Y
Zamanillo Campos (36)	Y	Y	Y	Y	Y	Y	Y	Y	Y	Y

Y, Yes; N, No; U, Unclear; NA, Not Applicable.

**Table 3:** Quality appraisal of included literatures using JBI checklist for randomized controlled trials (13 items, 4 literatures).

References	Q1	Q2	Q3	Q4	Q5	Q6	Q7	Q8	Q9	Q10	Q11	Q12	Q13
Cui (22)	Y	Y	Y	N	N	U	Y	Y	N	Y	U	Y	Y
Yang (24)	Y	Y	Y	N	N	U	N	Y	N	Y	Y	Y	Y
Bowrey (26)	Y	Y	Y	N	N	Y	Y	Y	Y	Y	Y	Y	Y
Obling (32)	Y	Y	Y	N	N	N	Y	Y	Y	Y	Y	Y	Y

Y, Yes; N, No; U, Unclear; NA, Not Applicable.

**Table 4:** Quality appraisal of included literatures using JBI checklist for quasi-experimental studies (9 items, 2 literatures).

References	Q1	Q2	Q3	Q4	Q5	Q6	Q7	Q8	Q9
Ma (31)	Y	N	U	Y	Y	Y	Y	Y	Y
Zeng (38)	Y	N	U	Y	Y	Y	Y	Y	Y

Y, Yes; N, No; U, Unclear; NA, Not Applicable.

### 3.4 Changes in QoL at different follow-up time points

The pooled analysis demonstrated significant improvements in QoL at 1 month (SMD = 0.29, 95% CI: 0.04–0.54, *P* = 0.021), 2 months (SMD = 0.54, 95% CI: 0.16–0.91, *P* = 0.005), 3 months (SMD = 0.64, 95% CI: 0.09–1.20, *P* = 0.024), and 4 months (SMD = 0.61, 95% CI: 0.23–0.99, *P* = 0.001). No significant improvement was observed at 6 months (SMD = 0.09, 95% CI: −0.20–0.38, *P* = 0.514). Detailed results for each follow-up time point are presented in Figure 2.

**Figure 2:**
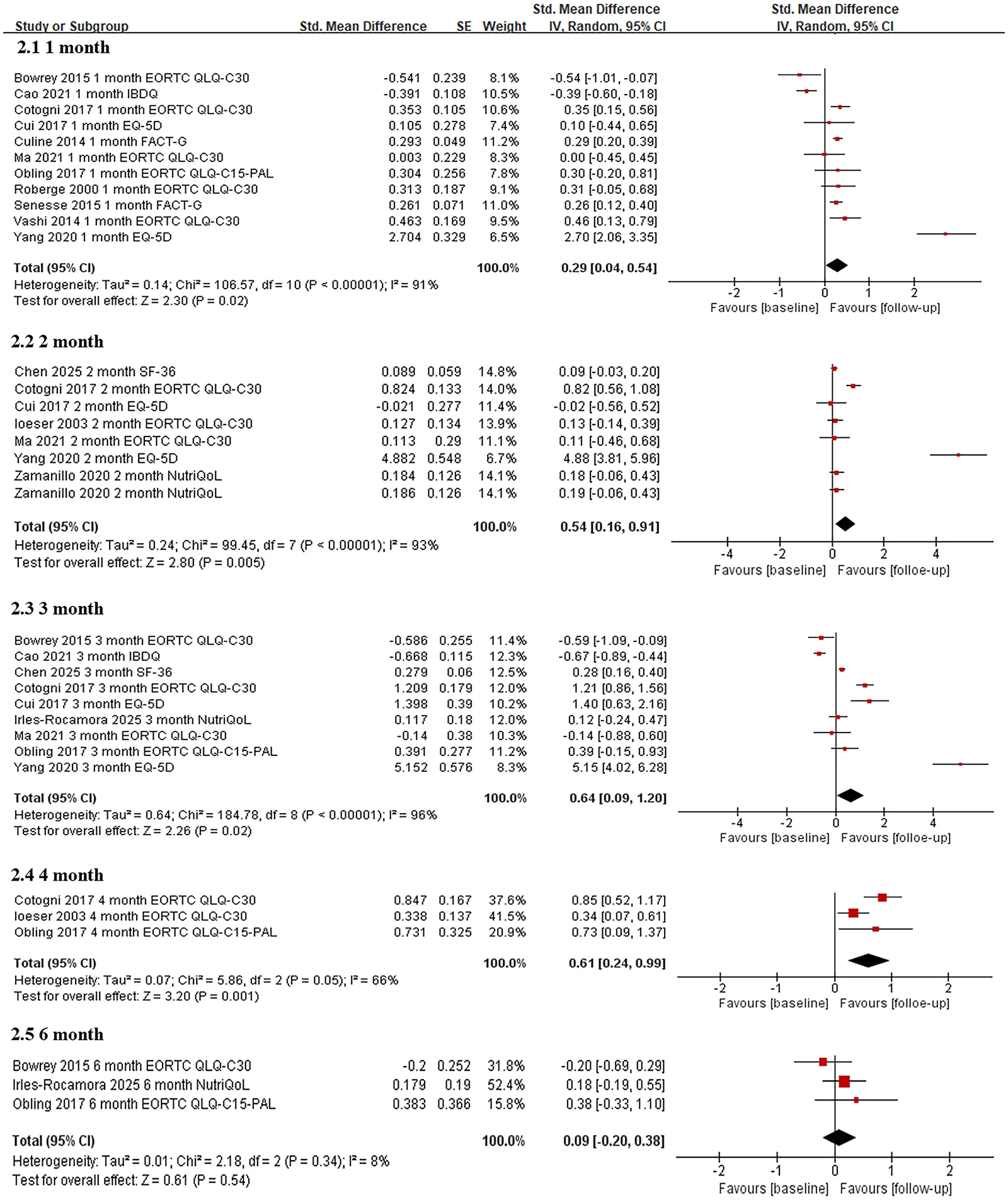
Forest plot of the meta-analysis for changes in quality of life among patients receiving home nutrition support at different follow-up time points.

### 3.5 Changes in QoL across follow-up time points

As shown in Figure 3, the pooled SMD estimates indicated statistically significant improvements from baseline in QoL at 1 to 4 months, with the largest pooled estimates observed at 2 to 4 months. The pooled estimate at 6 months was numerically smaller and did not reach statistical significance. The number of studies contributing to each time point ranged from 3 to 11, with high heterogeneity at most time points (*I*^2^: 8%−96%), supporting the use of random-effects models.

**Figure 3:**
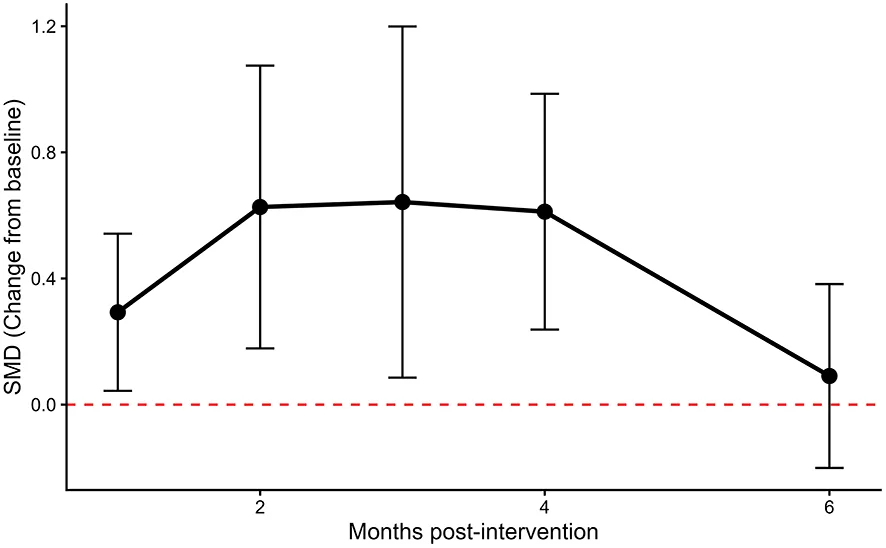
Changes in quality of life among patients receiving home nutrition support over time from baseline to 6 months post-intervention.

### 3.6 Descriptive domain-specific QoL outcomes

Three studies reported domain-specific QoL but were not included in the meta-analysis: Huang et al. (SF-36) and Zeng et al. (EORTC QLQ-C30) are shown in Figure 4, whereas Pinto-Sánchez et al. (SF-36) reported only change-from-baseline values (Δmean±SD) and are described narratively. In Figure 4, Huang et al. (*n* = 156) reported improvements in all eight SF-36 domains from discharge to 3 months, most notably physical functioning, mental health, and vitality; Zeng et al. (*n* = 30) reported improvements in EORTC QLQ-C30 functioning domains from 1 to 24 weeks after esophagectomy, particularly physical, social, and role function. Pinto-Sánchez et al. (*n* = 29) observed improvements in physical functioning, bodily pain, vitality, social functioning, and mental health at 1 and 3 months of HPN, with psychosocial domains improving more at 1 month and physical domains continuing to improve through 3 months.

**Figure 4:**
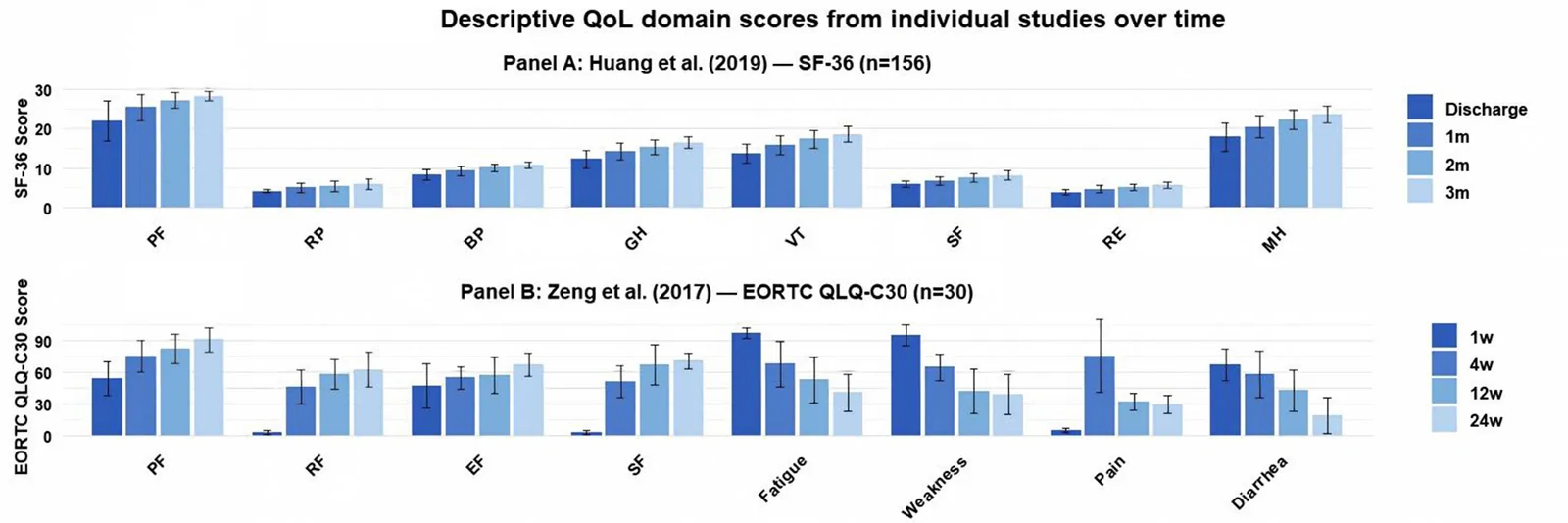
Descriptive QoL domain scores from individual studies over time. Huang et al. (23), patients with Crohn's disease, *n* = 156, SF-36; B: Zeng et al. (38), postoperative patients with esophageal cancer, *n* = 30, EORTC QLQ-C30. Bars represent means; error bars represent SD. SF-36: PF, physical functioning; RP, role-physical; BP, bodily pain; GH, general health; VT, vitality; SF, social functioning; RE, role-emotional; MH, mental health; higher scores indicate better status. EORTC QLQ-C30: PF, physical functioning; RF, role functioning; EF, emotional functioning; SF, social functioning; fatigue; Weakness; Pain; Diarrhea; higher scores on functional domains indicate better functioning, whereas higher scores on symptom domains indicate more severe symptoms. 1, 2, and 3m indicate 1, 2, and 3 months; 1, 4, 12, and 24w indicate 1, 4, 12, and 24 weeks.

### 3.7 Subgroup and sensitivity analyses

To investigate the substantial heterogeneity identified in the primary meta-analyses, subgroup analyses were further performed according to the type of HNS and the QoL measurement scales. The results showed that the mode of nutritional support (HEN/HPN) was not a significant source of between-study heterogeneity (see Supplementary Figure S1). Only within the subgroups of 1-month and 3-month follow-ups, the type of assessment scale represented a key factor driving heterogeneity in between-study effect sizes (*P* < 0.001). Moreover, certain subgroups exhibited persistently high heterogeneity (e.g., EQ-5D at 1 month, *I*^2^ = 97.3%), and several included only one or two studies, warranting particularly cautious interpretation. Detailed subgroup results are presented in Figure 5.

**Figure 5:**
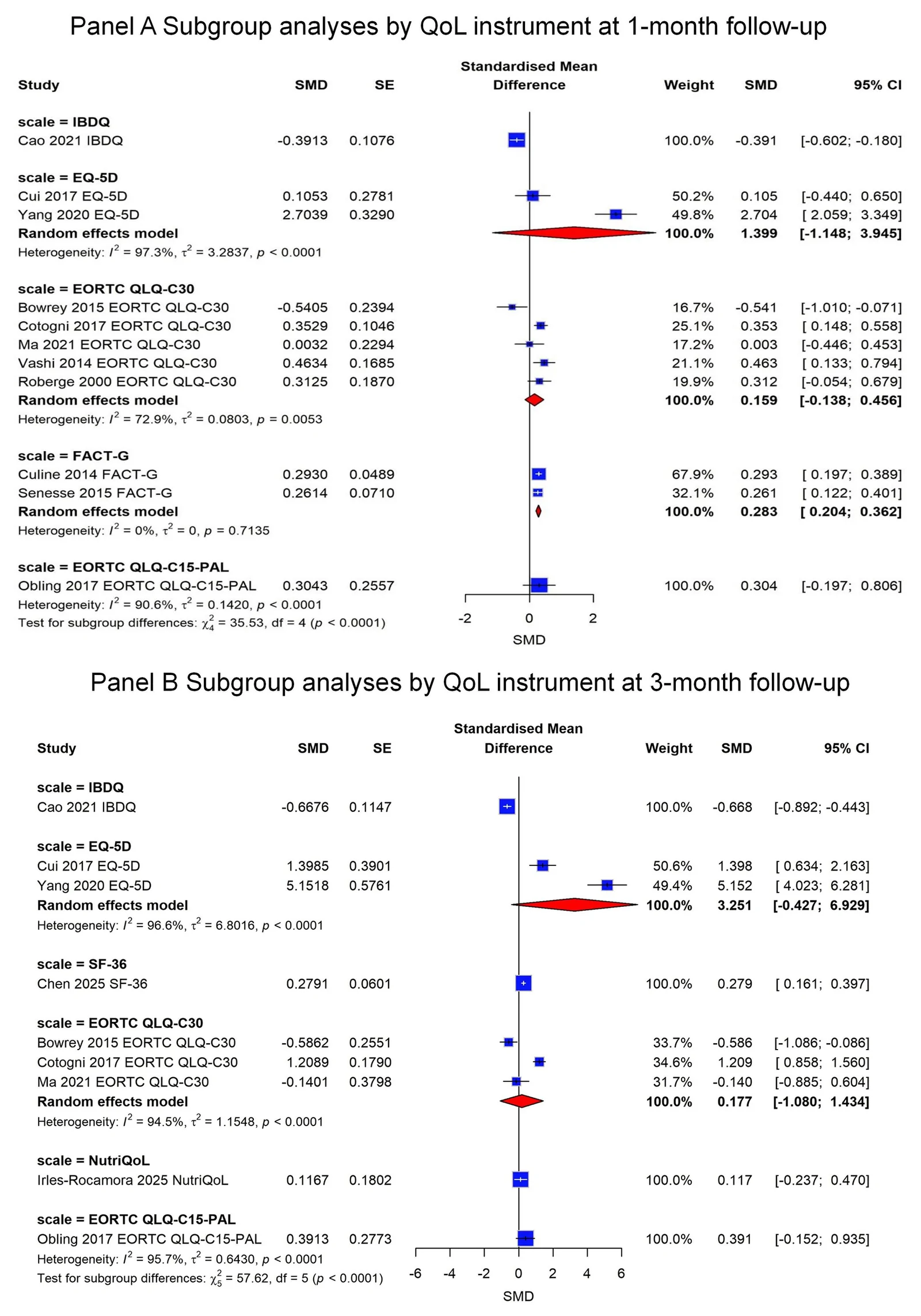
Subgroup analyses by QoL instrument at 1-month **(A)** and 3-month **(B)** follow-up.

Sensitivity analyses using alternative correlation coefficients (*r* = 0.10 and *r* = 0.90) showed similar temporal patterns to those observed in the primary analysis (*r* = 0.50). Although the magnitude of the pooled SMD estimates varied across assumptions, the estimates at 2 to 4 months were numerically larger than the estimate at 1 month, and the estimate at 6 months was numerically smaller; the 6-month estimate did not reach statistical significance in the primary analysis, indicating the robustness of the findings. Detailed Sensitivity analyses results are presented in Figure 6.

**Figure 6:**
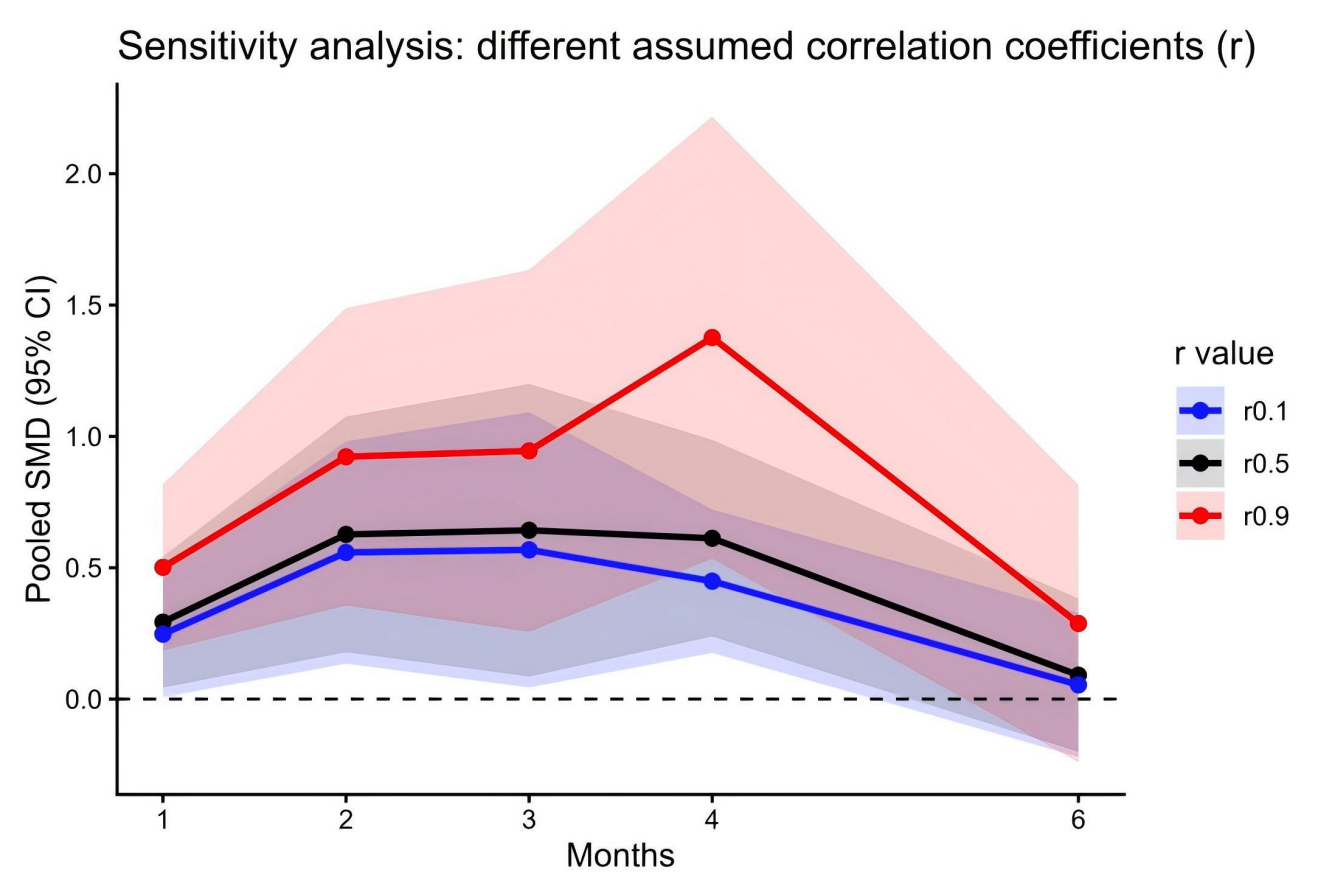
Sensitivity analysis: pooled SMD with different r values at multiple follow-up time points.

## 4 Discussion

### 4.1 Principal findings

To our knowledge, this is the first study to systematically evaluate the longitudinal changes of QoL in patients receiving HNS using a meta-analytic approach. Previous systematic reviews have primarily focused on the clinical effectiveness, safety, and impact of specific interventions on QoL (39–42), with evidence regarding changes in QoL over time largely confined to single time point comparisons or narrative summaries. In contrast, our study synthesized data across multiple follow–up time points to describe the pattern of changes in QoL following HNS initiation. Our results suggest that QoL improved significantly during the first 1–4 months, with the greatest improvements observed between 2 and 4 months; however, the improvement was smaller at 6 months and no longer statistically significant. Descriptive evidence suggested improvements across multiple physical and psychosocial domains—particularly bodily pain, social functioning, and general health—although the limited number of domain-specific reports precluded quantitative synthesis.

### 4.2 Changes in QoL during the first 4 months

The improvements from baseline were statistically significant at all four time points. Several factors may explain this pattern. Most patients initiate HNS in a state of nutritional deterioration (e.g., weight loss, fatigue), and nutritional repletion has been shown to restore energy balance and preserve physical function (5), which we also confirmed in our previous longitudinal study (25). These physiological gains create the foundation for subsequent psychological adjustment. As patients gradually master tube feeding or infusion techniques and reestablish daily routines, self-efficacy improves and anxiety diminishes—a staged process consistent with Chronic illness Trajectory Model (43). Furthermore, our recent randomized controlled trial on staged video education for HEN patients further corroborates that facilitating this adaptive process can alleviate functional impairment, anxiety, and depression (44).

Qualitative evidence further supports this adaptive process. Fu et al. (9) found that patients commonly experienced “positive experiences” and “adjustment to a new normal” during the early phase; Winkler et al. (45) found that many patients perceive HNS as a lifeline and equate QoL with the ability to resume daily activities and re-engage in social life. Collectively, these findings indicate that early QoL improvement results from the combined effect of nutritional repletion and psychological adaptation.

### 4.3 QoL outcomes at 6 months

However, the 6-month estimate was derived from a smaller and different set of studies than the earlier time points, so direct comparisons across time points should be interpreted with caution. If this pattern represents a real change, several factors could explain the lower estimate.

First, the natural history of the underlying disease can determine the trajectory of QoL changes, and this clinical heterogeneity warrants careful differentiation. For patients with advanced cancer, disease progression itself may gradually overshadow the benefits derived from nutritional support (40). Cotogni et al. (27) observed that QoL in patients with advanced cancer plateaued or declined after 4 months of HPN, a pattern consistent with our findings. Vashi et al. (35) reported that the greatest QoL benefit occurred at 3 months of HPN, after which survival declined markedly. In contrast, for patients with benign conditions such as Crohn's disease, the QoL change likely follows a fundamentally different course, as over 80% of these patients survive for 5 years on HPN (46). This distinction is clinically critical: our pooled estimates aggregate across these heterogeneous populations, and the observed attenuation at 6 months may disproportionately reflect the high attrition of advanced cancer patients too ill to provide follow-up data, rather than a universal phenomenon.

Second, cumulative physical and psychological burdens on patients and caregivers may also contribute to this attenuation (47). For HEN patients—particularly those with head and neck cancer—the tube feeding device imposes specific psychosocial challenges: although physical tolerance is acceptable, psychological tolerance is poor, with two thirds of patients longing for tube removal and nearly one quarter avoiding public outings (37). For HPN patients, the burden profile differs but is equally consequential, catheter-related fears, sleep disruption from pump noises and nocturia, and restricted social participation are commonly reported, with over 70% of patients experiencing feelings of dependency and more than half limiting their travel (48). These burdens are not static; they accumulate over time as the novelty of home therapy wanes and the reality of long-term device dependence intensifies, progressively straining patients' psychological reserves and social networks. These burdens, coupled with the failure of existing care systems to dynamically adjust to evolving patient needs, may jointly erode early improvements. This pattern is also consistent with the descriptive findings from Pinto-Sánchez et al., in which SF-36 domain scores appeared higher at 1 month than at 3 months, potentially reflecting the transition from an early “honeymoon phase” after discharge to the accumulating burdens of long-term HPN. However, this observation derives from a single small pilot study and requires cautious interpretation.

Third, as treatment extends beyond the initial 3 to 4 months, patients' core needs shift from mere nutritional supplementation to social participation, role restoration, and autonomous living. However, current care models remain heavily focused on nutritional parameters and complication management, with insufficient attention to vocational rehabilitation, psychosocial counseling, or social support (49, 50). Kirk et al. (51) found that even among patients with maintained nutritional status, long-term HPN users (median 41 months) reported extremely low HRQoL, with social functioning and employment status emerging as significant correlates—underscoring that social disconnection severely constrains long-term wellbeing despite adequate nutrition. This attenuation may also reflect insufficient continuity of care after hospital discharge—a core function that home nutrition support is intended to fulfill. Indeed, proactive telephone follow-up has been shown to maintain HRQoL in HEN patients (52), suggesting that the decline observed at 6 months may stem from weakening care continuity over time rather than an inevitable consequence of the treatment itself.

The explanatory factors discussed above are clinically plausible but were not directly tested in this review; moreover, the within-group design cannot isolate the effect of HNS from concurrent treatments or disease progression. These factors should therefore be considered as hypotheses rather than validated mechanisms.

### 4.4 Implications for clinical practice

The observed temporal patterns offer hypothesis-generating considerations rather than direct clinical implications. The 1- to 4-month period, during which QoL improvements were most consistently observed, may provide a theoretical foothold for optimizing nutritional regimens, patient education, and self-management support; however, whether interventions initiated specifically during this period yield superior outcomes compared with earlier or later initiation remains unproven. The numerically smaller and non-significant pooled estimate at 6 months—though based on a smaller and different set of studies—may suggest that early improvements are not necessarily maintained without ongoing support, highlighting the need for long-term care strategies. To address this, drawing on the chronic illness trajectory framework, we tentatively suggest a phase-adjusted multidisciplinary approach as a working hypothesis (48, 49). Crucially, these phased proposals were not directly tested by the pooled estimates, and we explicitly refrain from claiming that this specific timing sustains QoL. At the systemic level, HNS care models might evolve toward integrated biopsychosocial paradigms, though the optimal composition and timing of such support remain to be determined (46, 47).

### 4.5 Heterogeneity, sensitivity, and robustness

The subgroup analysis should be considered exploratory due to the limited number of studies at each time point, making definitive conclusions difficult. While different measurement scales may contribute to methodological heterogeneity (53), this is inherently confounded with clinical heterogeneity. Most included studies enrolled cancer patients, whereas two focused on Crohn's disease. The change of QoL during HNS may differ fundamentally between these populations–cancer patients face progressive disease burden and treatment toxicity, whereas Crohn's disease follows a relapsing-remitting course with distinct psychosocial stressors. Because QoL tools are highly population-specific (e.g., IBDQ for IBD, EORTC for cancer), the observed subgroup differences are likely driven by the underlying disease and clinical setting rather than the instruments themselves. Furthermore, mixed or unreported primary diagnoses precluded assessment of whether the QoL trends were modified by the underlying disease.

Importantly, sensitivity analyses showed that regardless of the correlation coefficient assumption adopted, the overall direction of the SMD estimates remained consistent. This suggests that the observed temporal pattern is broadly consistent across different r values, though the magnitude of the effect estimates varied. The correlation coefficient is a variance-estimation assumption rather than a statistical model choice, and the sensitivity analysis should be interpreted as supporting consistency in direction rather than confirming a real clinical phenomenon.

## 5 Limitation

Several limitations should be acknowledged. First, some time points, particularly 6 months, included few studies, no data were available beyond 6 months, and the studies contributing to each time point differed without formal between-time-point comparisons; the observed temporal pattern should therefore be interpreted as descriptive. Between-study heterogeneity in QoL instruments, disease profiles, and HNS modalities, along with mixed or unreported diagnoses, limited disease-specific subgroup analyses and may reduce generalizability.

Second, because correlation coefficients and domain-specific QoL scores were unavailable, the primary analysis relied on an imputed correlation (*r* = 0.50); sensitivity analyses using extreme values (0.10 and 0.90) yielded consistent directions, supporting robustness. However, incomplete reporting of attrition and missing data in the single-arm studies may have introduced bias.

Third, restricting the search to English and Chinese publications may have introduced language bias and excluded relevant studies, particularly from European countries with established home nutrition systems. The predominance of Chinese studies (7/18, ≈40%) may further limit generalizability to healthcare systems with different home nutrition policies and care delivery models. Future reviews should include multilingual studies.

## 6 Conclusion

This meta–analysis suggests that, in patients receiving HNS, significant QoL improvements from baseline were observed at 1–4 months, with the largest effect estimates noted at 2–4 months; the estimate at 6 months did not reach statistical significance. As these findings derive from separate sets of studies at each time point, they should not be interpreted as evidence of a continuous trajectory of improvement, peaking, and decline. The observed changes may be consistent with hypotheses involving nutritional benefits, disease progression, treatment burden, and psychosocial adaptation, though our design cannot establish causation. Future research should employ rigorous prospective designs with extended follow–up and formal comparisons between time points, incorporate patient–reported outcomes alongside qualitative experiences, and explicitly test whether distinct QoL changes exist across patient subgroups to inform individualized support strategies.

## Data Availability

Publicly available datasets were analyzed in this study. The datasets used in this meta-analysis are extracted from previously published peer-reviewed studies. All required data have been presented in the tables within the article /Supplementary material.
