# Supplementary material for: Longitudinal changes in quality of life among patients receiving home nutrition support: a systematic review and meta-analysis
Source: Front Nutr. 2026 Oct 19;13:1940328. doi: 10.3389/fnut.2026.1940328 (PMC13724585; doi:10.3389/fnut.2026.1940328)
Supplement: Supplementary file 1 [file Table_1.docx]

3 Supplementary Material

# 1 Supplementary Text S1

Complete search strategies for all databases (PubMed, Embase, Web of Science, CINAHL, Cochrane Library, CNKI, Wanfang, VIP, and SinoMed). Searched from inception to June 1, 2026.

PubMed

("Parenteral Nutrition, Home"[Mesh] OR "home enteral nutrition"[tiab] OR "home parenteral nutrition"[tiab] OR "home nutritional support"[tiab] OR "home tube feeding"[tiab] OR "home enteral tube feeding"[tiab]) AND ("Quality of Life"[MeSH] OR "quality of life"[tiab] OR "QoL"[tiab] OR "QOL"[tiab] OR "health related quality of life"[tiab] OR "HRQoL"[tiab] OR "HRQL"[tiab] OR "life quality"[tiab])

Embase

('home parenteral nutrition'/exp OR 'home enteral nutrition':ti,ab,kw OR 'home parenteral nutrition':ti,ab,kw OR 'home nutritional support':ti,ab,kw OR 'home tube feeding':ti,ab,kw) AND ('quality of life'/exp OR 'health related quality of life':ti,ab,kw OR 'hrqol':ti,ab,kw OR 'life quality':ti,ab,kw)

Web of Science

TS=("home enteral nutrition" OR "home parenteral nutrition" OR "home nutritional support" OR "home tube feeding" OR "home enteral tube feeding") AND TS=("quality of life" OR "QoL" OR "QOL" OR "health related quality of life" OR "HRQoL" OR "HRQL" OR "life quality")

Cochrane Library

#1 ("home enteral nutrition" OR "home parenteral nutrition" OR "home nutritional support" OR "home tube feeding" OR "home enteral tube feeding"):ti,ab,kw

#2 ("quality of life" OR "QoL" OR "QOL" OR "health related quality of life" OR "HRQoL" OR "HRQL" OR "life quality"):ti,ab,kw

#3 #1 AND #2

CNKI（知网）

SU='家庭营养' OR SU='家庭肠内营养' OR SU='家庭肠外营养' OR SU='口服营养补充' OR SU='管饲' AND (SU='生活质量' OR SU='健康相关生活质量' OR SU='患者生活质量' OR SU='生存质量' OR SU='QOL')

Wan fang（万方）

主题:(家庭营养支持 OR 家庭肠内营养 OR 家庭肠外营养 OR 口服营养补充 OR 管饲) AND 主题:(生活质量 OR 健康相关生活质量 OR 患者生活质量 OR 生存质量 OR QOL)

VIP（维普）

M=(家庭营养支持 OR 家庭肠内营养 OR 家庭肠外营养 OR 口服营养补充 OR 管饲) AND M=(生活质量 OR 健康相关生活质量 OR 生存质量 OR 患者生活质量 OR QOL)

SinoMed（中国生物医学文献数据库）

(家庭营养支持[常用字段:智能] OR 家庭肠内营养[常用字段:智能] OR 家庭肠外营养[常用字段:智能] OR 口服营养补充[常用字段:智能] OR 管饲[常用字段:智能]) AND (生活质量[常用字段:智能] OR 健康相关生活质量[常用字段:智能] OR 生存质量[常用字段:智能] OR 患者生活质量[常用字段:智能] OR QOL[常用字段:智能]) AND (家庭[常用字段:智能] OR 居家[常用字段:智能])

# 2 Supplementary Table S1

JBI Critical Appraisal Checklist Items (Chinese and English Translation). Source: https://jbi.global/critical-appraisal-tools.

## 2.1 Supplementary Table S1a. JBI Critical Appraisal Checklist for Randomized Controlled Trials (13 items)

| **Item** | **English Criteria** | **中文条目** |
| --- | --- | --- |
| Q1 | Was true randomization used for assignment of participants to treatment groups? | 研究对象分配是否真正采取了随机化分组？ |
| Q2 | Was allocation to treatment groups concealed? | 分组方案是否采取了分配隐藏？ |
| Q3 | Were treatment groups similar at baseline? | 试验组和对照组基线是否具有可比性？ |
| Q4 | Were participants blind to treatment assignment? | 是否对研究对象采取了盲法？ |
| Q5 | Were those delivering treatment blind to treatment assignment? | 是否对干预者采取了盲法？ |
| Q6 | Were outcome assessors blind to treatment assignment? | 是否对结果测评者采取了盲法？ |
| Q7 | Were treatment groups treated identically other than the intervention of interest? | 除了要验证的干预措施，各组接受的其他措施是否相同？ |
| Q8 | Was follow-up complete and if not, were differences between groups in terms of their follow-up adequately described and analyzed? | 随访是否完整，如不完整，是否采取措施处理？ |
| Q9 | Were participants analyzed in the groups to which they were randomized? | 是否将所有入组的研究对象均纳入结果分析中？ |
| Q10 | Were outcomes measured in the same way for treatment groups? | 是否采用相同的方式对各组研究对象的结局指标进行测评？ |
| Q11 | Were outcomes measured in a reliable way? | 结局指标的测评方法是否可信？ |
| Q12 | Was appropriate statistical analysis used? | 资料分析方法是否恰当？ |
| Q13 | Was the trial design appropriate, and any deviations from the standard RCT design (individual randomization, parallel groups) accounted for in the conduct and analysis of the trial? | 研究设计是否合理？在研究实施和分析过程中是否有偏离标准RCT之处？ |

## 2.2 Supplementary Table S1b. JBI Critical Appraisal Checklist for Case Series (10 items)

| Item | English Criteria | 中文条目 |
| --- | --- | --- |
| Q1 | Were there clear criteria for inclusion in the case series? | 是否明确了病例系列的纳入标准？ |
| Q2 | Was the condition measured in a standard, reliable way for all participants? | 是否采用标准、可靠的方式对所有参与者进行疾病状态测评？ |
| Q3 | Were valid methods used for identification of the condition for all participants? | 是否采用有效的方法对所有参与者进行疾病状态识别？ |
| Q4 | Did the case series have consecutive inclusion of participants? | 是否连续纳入参与者？ |
| Q5 | Did the case series have complete inclusion of participants? | 是否完整纳入了所有符合条件的参与者？ |
| Q6 | Was there clear reporting of the demographics of the participants in the study? | 是否清晰报告了参与者的人口学特征？ |
| Q7 | Was there clear reporting of clinical information of the participants? | 是否清晰报告了参与者的临床信息？ |
| Q8 | Were the outcomes or follow-up results of cases clearly reported? | 是否清晰报告了病例的结局或随访结果？ |
| Q9 | Was there clear reporting of the presenting site(s)/clinic(s) demographic information? | 是否清晰报告了就诊地点/诊所的人口学信息？ |
| Q10 | Was statistical analysis appropriate? | 资料分析方法是否恰当？ |

## 2.3 Supplementary Table S1c. JBI Critical Appraisal Checklist for Quasi-Experimental Studies (9 items)

| Item | English Criteria | 中文条目 |
| --- | --- | --- |
| Q1 | Is it clear in the study what is the 'cause' and what is the 'effect' (i.e. there is no confusion about which variable comes first)? | 研究是否明确了因果关系的方向（即不存在变量先后顺序的混淆）？ |
| Q2 | Were the participants included in any comparisons similar? | 参与者在各组间的可比性如何？ |
| Q3 | Were the participants included in any comparisons receiving similar treatment/care, other than the exposure or intervention of interest? | 除了待研究的暴露或干预措施外，各组参与者是否接受了相似的治疗/护理？ |
| Q4 | Was there a control group? | 是否有对照组？ |
| Q5 | Were there multiple measurements of the outcome both pre and post the intervention/exposure? | 是否在干预/暴露前后对结局进行了多次测量？ |
| Q6 | Was follow-up complete and if not, were differences between groups in terms of their follow-up adequately described and analyzed? | 随访是否完整，如不完整，是否采取措施处理？ |
| Q7 | Were the outcomes of participants included in any comparisons measured in the same way? | 是否采用相同的方式对各组参与者的结局指标进行测评？ |
| Q8 | Were outcomes measured in a reliable way? | 结局指标的测评方法是否可信？ |
| Q9 | Was appropriate statistical analysis used? | 资料分析方法是否恰当？ |

# 3 Supplementary Table S2

## 3.1 Summary of excluded studies due to insufficient QoL data (n = 44)

| First author, year | QoL instrument | Missing data |
| --- | --- | --- |
| Li 2015 | IBDQ | No fixed follow-up time; only reported as "mean 148 d (range 29–312 d)" — timing of post-assessment not standardized; |
| Cai L, 2019 | EORTC QLQ-C30 + QLQ-STO22 | No baseline QoL data reported — only post-intervention scores at 1 month were compared between groups; |
| Zhang WQ, 2016 | None | No QoL assessment was performed. |
| Gao P, 2020 | EORTC QLQ-C30 | QoL outcomes reported as median (IQR) only, not mean±SD. |
| Jiang Y, 2019 | QOL score (unspecified scale) | Baseline QoL data not reported — only post-intervention scores compared between groups; |
| Shi 2020 | None | No QoL assessment was performed. |
| Ren HM, 2018 | EORTC QLQ-C30 | Baseline QoL data not reported. |
| Wang Q 2019 | EORTC QLQ-C30 | Baseline QoL data not reported. |
| Xu XY, 2017 | KPS | KPS is a performance status score, not a patient-reported QoL instrument. |
| Wang LJ, 2018 | None | No QoL assessment was performed. |
| Cang YZ, 2016 | KPS used for performance status | No standardized QoL instrument used. KPS (performance status) is not a QoL questionnaire |
| Wang Y, 2023 | None | No QoL assessment was performed. |
| Wang ZM 2007 | KPS used for performance status | No standardized QoL instrument used. KPS (performance status) is not a QoL questionnaire |
| Aeberhard C, 2015 | SF-36 | No total QoL score reported; subscale scores only partially reported. |
| DiMartini A, 1998 | QOLI | No total QoL score reported. |
| Baker ML, 2017 | None | No QoL assessment was performed. |
| Bonifacio R, 2007 | Karnofsky Index (KI) | No mean±SD or median (IQR) values reported for KI scores. |
| Bozzetti F, 2002 | Rotterdam Symptom Checklist (RSCL) | No numerical QoL scores (e.g., mean±SD or median) reported in the abstract or available summary. |
| Chambers A, 2006 | SF-36 (PCS, MCS), EQ-5D, HADS | Standard deviations (SD) or standard errors (SE) not reported for any QoL score. |
| Bouleuc C, 2020 | EORTC QLQ-C15-PAL | No numerical QoL scores (mean±SD or median) at specific time points were reported. |
| Cotogni P, 2020 | None | No QoL assessment was performed. |
| Donohoe CL, 2017 | EORTC QLQ-C30 + QLQ-OES18 | Standard deviations (SD) not reported in the available abstract/full text. |
| Fan KH, 2025 | None | No QoL assessment was performed. |
| Li Z, 2019 | None | No baseline QoL data reported. |
| Girke J, 2016 | EORTC QLQ-C30 | No numerical QoL scores (mean±SD or median/IQR) reported. |
| King LA, 1993 | EORTC QLQ-C30 | No numerical QoL scores (mean±SD or median/IQR) reported. |
| Li W, 2022 | EORTC QLQ-C15-PAL | Duplicate publication. Data derived from an already included RCT (Obling et al., 2017; same patient cohort). |
| Lucchin L, 2012 | Self-designed 16-item questionnaire | QoL assessed in caregivers, not patients |
| Paccagnella A, 2007 | SF-12 | No specific QoL scores reported; only qualitative conclusions provided |
| Pironi L, 2004 | SF-36 | No numerical QoL scores (mean±SD or median/IQR) reported. |
| Richter E, 2012 | None (non-standardized) | no numerical QoL scores (mean±SD or median/IQR) reported. |
| Ruggeri E, 2022 | KPS used for performance status | No standardized QoL instrument used. KPS (performance status) is not a QoL questionnaire |
| Schönenberger KA, 2023 | SF-36 | QoL data complete (standardized instrument, baseline and follow-up, mean±SD). |
| Lee A, 2021 | SF-36 | no numerical QoL scores (mean±SD or median/IQR) reported for any SF-36 domain. |
| Jones D, 2023 | PNIQ | No mean±SD or median (IQR) of PNIQ scores at baseline or follow-up time points reported. |
| Lacoste C, 2024 | NUTRIQoL | No baseline QoL data reported (only post-discharge assessments); data reported as median (IQR) not mean±SD; |
| Qian Z, 2014 | EORTC QLQ-C30 | No baseline QoL data reported; no standard deviations (SD) provided for any QoL score; EORTC QLQ-C30 scores not reported numerically. |
| Rovera GM, 1998 | QOLI | No numerical QoL scores (mean±SD or median) reported for any of the 25 domains; no total QoL score provided. |
| Stanner H, 2022 | SF-36 | No numerical QoL scores (mean±SD or median) reported for any of the 25 domains; no total QoL score provided. |
| Stanga Z, 2016 | SF-36 | No standard deviations (SD) or standard errors (SE) reported for any QoL score. |
| Malone M, 2002 | SF-36 | No numerical QoL scores (mean±SD or median) reported for any SF-36 domain. |
| Li FX, 2024 | None | No QoL assessment was performed. |
| Liu L, 2018 | None | No QoL assessment was performed. |
| Liu XL, 2020 | None | No QoL assessment was performed. |

Note: QoL, quality of life; SD, standard deviation; SE, standard error; IQR, interquartile range; KPS, Karnofsky Performance Status; IBDQ, Inflammatory Bowel Disease Questionnaire; EORTC QLQ-C30, European Organization for Research and Treatment of Cancer Quality of Life Questionnaire Core 30; EORTC QLQ-STO22, EORTC Quality of Life Questionnaire Stomach Cancer Module; EORTC QLQ-OES18, EORTC Quality of Life Questionnaire Oesophageal Cancer Module; EORTC QLQ-C15-PAL, EORTC Quality of Life Questionnaire Core 15 Palliative; FACT-G, Functional Assessment of Cancer Therapy-General; SF-36, Short Form 36; SF-12, Short Form 12; EQ-5D, EuroQol 5 Dimensions; HADS, Hospital Anxiety and Depression Scale; QOLI, Quality of Life Instrument; PNIQ, Parenteral Nutrition Impact Questionnaire; RSCL, Rotterdam Symptom Checklist; NUTRIQoL, Nutrition Quality of Life Questionnaire; PCS, Physical Component Summary; MCS, Mental Component Summary. Detailed reasons for exclusion are provided in the "Reason for exclusion" column.

References of excluded studies（n=44）

（1）Li P, Wang X, Peng N, et al. Implementation and effect of home enteral nutrition support in outpatients with Crohn's disease. Chinese Journal of Clinical Nutrition. 2015;23(6):378-381. (in Chinese) doi: 10.3760/cma.j.issn.1674-635X.2015.06.010
（2）Cai L, Yang Y, He XL. Effect of oral nutritional supplementation therapy on quality of life in gastric cancer patients during chemotherapy. Guangdong Vocational Technical Education and Research. 2019;(5):174-176. (in Chinese) doi: 10.19494/j.cnki.issn1674-859x.2019.05.060
（3）Zhang WQ, Huang JL, Lin DL, et al. Observation on the therapeutic effect of home enteral nutrition after esophagectomy. Public Medical Forum Magazine. 2016;20(34):4774-4776. doi: 10.19435/j.1672-1721.2016.34.007
（4）Gao Peng, Wang Nan, Wu Di, et al. Effect of enteral nutrition on short-term nutritional status and quality of life in postoperative patients with gastric cancer. Chinese Journal of Endocrine Surgery. 2020;14(6):445-449. (in Chinese) doi: 10.3760/[cma.j.cn](https://cma.j.cn).115807-20200826-00257
（5）Jiang Y, Meng QY, Tan SJ, et al. Prospective randomized controlled clinical study of the effects of oral nutritional supplementation on body weight and quality of life in patients with gastric cancer and colorectal cancer after discharge. Chinese Journal of Clinical Nutrition. 2019;27(5):271-275. doi: 10.3760/cma.j.issn.1674-635X.2019.05.002
（6）Shi HY, Chen HL, Ding EH. Analysis of influencing factors and countermeasures of oral nutritional supplement for patients discharged from hospital after radical esophagectomy. China Modern Doctor. 2020;58(22):162-165. (in Chinese)
（7）Ren Huimin, Su Zhuobin, Yang Yongjiang, et al. Feasibility study of home enteral nutrition in patients after total gastrectomy for gastric cancer. Food and Nutrition in China. 2018;24(10):61-63. (in Chinese)
（8）Wang Q, Wang ZH, Wang P, et al. Effect of home enteral nutrition support therapy on nutritional status of patients after esophagectomy for esophageal cancer. Journal of Clinical Research. 2019;36(8):1651-1652. (in Chinese) doi: 10.3969/j.issn.1671-7171.2019.08.077
（9）Xu XY. Evaluation of the efficacy of home enteral nutrition in postoperative patients with gastric cancer [Master's thesis]. Changchun: Jilin University, 2015. (in Chinese)
（10）Wang Lijuan, Yang Xi, Chen Xu, et al. Effect of oral nutritional supplementation on nutritional status and pulmonary function in elderly patients with stable chronic obstructive pulmonary disease after discharge. China Modern Doctor. 2018;56(20):119-122. (in Chinese)
（11）Cang Yuzhen, Zeng Qingshan, Huang Wenjing. Effect of early enteral nutrition intervention on nutritional indicators in discharged patients after gastric cancer surgery. Hainan Medical Journal. 2016;27(19):3170-3172. (in Chinese) doi: 10.3969/j.issn.1003-6350.2016.19.025
（12）Wang Yu, Zhao Qing, Luo Yongbing, et al. Clinical study of oral nutritional supplement intervention improving protein-energy wasting in hemodialysis patients. Chinese Journal of Blood Purification. 2023;22(1):11-16. (in Chinese)
（13）Wang ZM, Jiang ZW, Zou ZY, et al. Application of percutaneous endoscopic gastrostomy/jejunostomy in patients receiving home enteral nutrition support. Journal of Medical Postgraduates. 2007;(11):1123-1125. (in Chinese) doi: 10.16571/j.cnki.1008-8199.2007.11.004
（14）Aeberhard C, Leuenberger M, Joray M, Ballmer PE, Mühlebach S, Stanga Z. Management of Home Parenteral Nutrition: A Prospective Multicenter Observational Study. Ann Nutr Metab. 2015;67(4):210-217. doi:10.1159/000440683
（15）DiMartini A, Rovera GM, Graham TO, et al. Quality of life after small intestinal transplantation and among home parenteral nutrition patients. JPEN J Parenter Enteral Nutr. 1998;22(6):357-362. doi:10.1177/0148607198022006357
（16）Baker ML, Halliday V, Robinson P, Smith K, Bowrey DJ. Nutrient intake and contribution of home enteral nutrition to meeting nutritional requirements after oesophagectomy and total gastrectomy. Eur J Clin Nutr. 2017;71(9):1121-1128. doi:10.1038/ejcn.2017.88
（17）Bonifacio R, Alfonsi L, Santarpia L, et al. Clinical outcome of long-term home parenteral nutrition in non-oncological patients: a report from two specialised centres. Intern Emerg Med. 2007;2(3):188-195. doi:10.1007/s11739-007-0056-4
（18）Bozzetti F, Cozzaglio L, Biganzoli E, et al. Quality of life and length of survival in advanced cancer patients on home parenteral nutrition. Clin Nutr. 2002;21(4):281-288. doi:10.1054/clnu.2002.0560
（19）Chambers A, Hennessy E, Powell-Tuck J. Longitudinal trends in quality of life after starting home parenteral nutrition: a randomised controlled study of telemedicine. Clin Nutr. 2006;25(3):505-514. doi:10.1016/j.clnu.2006.01.001
（20）Alderman B, Allan L, Amano K, et al. Multinational Association of Supportive Care in Cancer (MASCC) expert opinion/guidance on the use of clinically assisted nutrition in patients with advanced cancer. Support Care Cancer. 2022;30(4):2983-2992. doi:10.1007/s00520-021-06613-y
（21）Bouleuc C, Anota A, Cornet C, et al. Impact on health-related quality of life of parenteral nutrition for patients with advanced cancer cachexia: results from a randomized controlled trial. Oncologist. 2020;25(5):e843-e851. doi: 10.1634/theoncologist.2019-0566
（22）Cotogni P, Monge T, Passera R, Brossa L, De Francesco A. Clinical characteristics and predictive factors of survival of 761 cancer patients on home parenteral nutrition: A prospective, cohort study. Cancer Med. 2020;9(13):4686-4698. doi:10.1002/cam4.3064
（23）Donohoe CL, Healy LA, Fanning M, et al. Impact of supplemental home enteral feeding postesophagectomy on nutrition, body composition, quality of life, and patient satisfaction. Dis Esophagus. 2017;30(9):1-9. doi:10.1093/dote/dox063
（24）Fan K, Patel P, Maddison R, McKenzie E, Wilson N, Massey D, et al. Evaluation of survival outcomes in patients receiving palliative home parenteral nutrition: results from a UK tertiary referral centre. United European Gastroenterol J. 2025;13:1008. doi: 10.1002/ueg2.70036
（25）Li Z, Li L, Wang SB. Prospective study on the effects of oral nutritional supplementation on nutritional status and immune function in patients with gastrointestinal malignant tumors during adjuvant chemotherapy after radical resection. Journal of Basic and Clinical Oncology. 2019;32(5):443-445. (in Chinese)
（26）Girke J, Seipt C, Markowski A, et al. Quality of Life and Nutrition Condition of Patients Improve Under Home Parenteral Nutrition: An Exploratory Study. Nutr Clin Pract. 2016;31(5):659-665. doi:10.1177/0884533616637949
（27）King LA, Carson LF, Konstantinides N, et al. Outcome assessment of home parenteral nutrition in patients with gynecologic malignancies: what have we learned in a decade of experience?. Gynecol Oncol. 1993;51(3):377-382. doi:10.1006/gyno.1993.1307
（28）Li W, Guo H, Li L, Cui J. Cost-Effectiveness Analyses of Home Parenteral Nutrition for Incurable Gastrointestinal Cancer Patients. Front Oncol. 2022;12:858712. doi: 10.3389/fonc.2022.858712
（29）Lucchin L, Vedovato L, Schrei M, Kob M. Caregivers in the management of home-enteral-nutrition-patients: observational study on epidemiology, organisation, technical aspects and quality of life. Mediterranean Journal of Nutrition and Metabolism. 2012;5(3):233-239. doi: 10.1007/s12349-011-0077-4
（30）Paccagnella A, Mauri A, Berto R, et al. Biopsychosocial approach to home enteral nutrition: measure of subjective satisfaction and quality of life. Minerva Med. 2007;98(1):5-17.
（31）Pironi L, Paganelli F, Mosconi P, et al. The SF-36 instrument for the follow-up of health-related quality-of-life assessment of patients undergoing home parenteral nutrition for benign disease. Transplant Proc. 2004;36(2):255-258. doi:10.1016/j.transproceed.2003.12.008
（32）Richter E, Denecke A, Klapdor S, Klapdor R. Parenteral nutrition support for patients with pancreatic cancer--improvement of the nutritional status and the therapeutic outcome. Anticancer Res. 2012;32(5):2111-2118.
（33）Schönenberger KA, Reber E, Huwiler VV, et al. Quality of Life in the Management of Home Parenteral Nutrition. Ann Nutr Metab. 2023;79(3):326-333. doi:10.1159/000530082
（34）Lee A, Rocha M, Gomes D, Pereira J, Barbosa V, Yano O, et al. Quality of life assessment of the home parenteral nutrition patients in the waiting list for intestinal transplantation in Brazil [Abstract]. Transplantation. 2021;105(7S):S99. doi: 10.1097/[01.tp](https://01.tp).0000758184.46064.c0
（35）Jones D, Lal S, French C, et al. Investigating the Relationship between Home Parenteral Support and Needs-Based Quality of Life in Patients with Chronic Intestinal Failure: A National Multi-Centre Longitudinal Cohort Study. Nutrients. 2023;15(3):622. Published 2023 Jan 25. doi:10.3390/nu15030622
（36）Lacoste C, Vernier T, Premaud K, et al. Assessment of quality of life using the NUTRIQoL® score in patients on enteral nutrition at 1 and 3 months of hospital discharge [Abstract]. Clin Nutr ESPEN. 2025;69:927-928. doi: 10.1016/j.clnesp.2025.07.339
（37）Qian Z, Sun Y, Ye Z, et al. Application of home enteral nutrition and its impact on the quality of life in patients with advanced gastric cancer. Chinese Journal of Gastrointestinal Surgery. 2014;17(2).
（38）Rovera GM, DiMartini A, Schoen RE, Rakela J, Abu-Elmagd K, Graham TO. Quality of life of patients after intestinal transplantation. Transplantation. 1998;66(9):1141-1145. doi:10.1097/00007890-199811150-00005
（39）Stanner H, Hamilton K, DeLegge M, Kristofek L. Quality of life measurements over 24 months in patients receiving home parenteral nutrition [Abstract]. JPEN J Parenter Enteral Nutr. 2023;47:S206-S208. doi: 10.1002/jpen.2491
（40）Stanga Z, Aeberhard C, Schärer P, Kocher A, Adler S, Villiger PM. Home parenteral nutrition is beneficial in systemic sclerosis patients with gastrointestinal dysfunction. Scand J Rheumatol. 2016;45(1):32-35. doi:10.3109/03009742.2015.1064543
（41）Malone M. Longitudinal assessment of outcome, health status, and changes in lifestyle associated with long-term home parenteral and enteral nutrition. JPEN J Parenter Enteral Nutr. 2002;26(3):164-168. doi:10.1177/0148607102026003164
（42）Li FX, Chen CY. Establishment and application of a nursing pathway for home enteral nutrition support with a nasojejunal tube after esophageal cancer surgery. Health World. 2024;(12):111-112. (in Chinese)
（43）Liu L, Qi C, Yu H. Effect of home enteral nutrition support on postoperative malnutrition patients with esophageal cancer. Family Medicine. Medical Treatment and Drug Selection. 2018;(8):121-122. (in Chinese)
（44）Liu Xiaolin, Jiang Ming, Jia Hui, et al. Analysis of the therapeutic effect of home enteral nutrition in postoperative patients with esophageal cancer. Anti-Tumor Pharmacy. 2020;10(1):112-115,128. (in Chinese) doi: 10.3969/j.issn.2095-1264.2020.01.21

# 4 Supplementary Table S3

## 4.1 Additional quality appraisal parameters for the 12 single‑arm studies: attrition rates, handling of missing data, and confounding control

| Study | Attrition rate | Handling of missing data | Confounding control |
| --- | --- | --- | --- |
| Cao 2021 | 27.34%（35/128） | Not reported | Not reported |
| Huang 2019 | 0%（0/156） | Not reported | Not reported |
| Chen 2025 | 0%（0/288） | LMM for random missing; complete-case analysis | Multivariate regression (14 variables) |
| Cotogni 2017 | 0%（0/111） | LMM for repeated measures (handles random missing) | Univariate mixed-effects models (8 covariates) |
| Culine 2014 | 41.7%（330/767） | Not reported | Partial: multivariate logistic regression for PS improvement only; QoL main analysis unadjusted |
| Irles-Rocamora 2025 | 36.8%（21/57） | Complete-case analysis; no imputation | Not reported |
| Loeser 2003 | 71%（40/56） | Not reported | Partial: multivariate regression in Study 1 only; Study 2 unadjusted |
| Pinto-Sánchez 2019 | 1 mo: 27.6%（8/29）; 3 mo: 37.9%（11/29） | Mean substitution (<10%); multiple imputation (>10%) | Not reported (subgroup analysis only) |
| Roberge 2000 | 23.1%（9/39） | Not reported | Not reported |
| Senesse 2015 | 20.3%（57/281） | Not reported | Partial: GLM/ANOVA for PS-nutrition association only; QoL main analysis unadjusted |
| Vashi 2014 | 25.0%（13/52 | GEE | Multivariate GEE (age, gender, treatment history) |
| Zamanillo 2020 | 17.9%（14/78） | Not reported | Binary logistic regression (adjusted for age and sex) |

Abbreviations: LMM, linear mixed model; GEE, generalized estimating equations; GLM, general linear model; ANOVA, analysis of variance; PS, performance status; QoL, quality of life. Data are presented as attrition rate (number lost to follow‑up / total number enrolled). “Not reported” indicates that the study did not describe the method for handling missing data or did not perform confounding adjustment. Chen 2025 and Cotogni 2017 reported 0% attrition because all enrolled patients were followed up; however, Chen 2025 excluded patients with incomplete data, which may have introduced selection bias. Studies with partial confounding control performed multivariable analyses only for secondary outcomes (e.g., PS improvement) but not for the primary QoL analysis.

# 5 Supplementary Figure S1

**Figure S1：Subgroup analyses by type of home nutrition support (HEN vs. HPN)**

**Supplementary Figure S1A：1month**

**Supplementary Figure S1B：2 month**

**Supplementary Figure S1C：3 month**

**Supplementary Figure S1D：4 month**

**Supplementary Figure S1E：6 month**
